# Agrochemical innovation for crop health: moving forward through dynamic disorder

**DOI:** 10.3389/fpls.2025.1719658

**Published:** 2026-01-09

**Authors:** An-Shan Hsiao

**Affiliations:** School of Biosciences, University of Birmingham, Birmingham, United Kingdom

**Keywords:** agrochemical, RNA interference, antimicrobial peptides, nanotechnology, intrinsically disordered proteins/regions, biomolecular condensates, sustainable agriculture

## Abstract

Global climate change has huge negative impacts on crop health. Strategies for increasing crop yield and resistance to biotic and abiotic stress factors (pathogenic microbes, insect pests, drought and heat waves, flooding etc.) are important to achieve sustainable agriculture for food security. Breeding for elite crop varieties takes time and may face challenges in commercialization. Besides the development of genetic tools to generate climate-smart crops, recent innovations in agrochemicals as interventions for improving crop health are emerging. This article discusses the current advances in three small technologies, RNAs, peptides and nanotechnology, in targeting plant diseases and improving productivity as well as in future research directions for agrochemical innovation. Although previously assumed as undruggable in human diseases, protein disorder has unique conformational features and plays critical roles in regulating various biological processes controlling crop productivity and stress resistance *in planta*. The article proposes the potential incorporation of protein disorder in these three approaches and intelligent agrochemical innovation with tailored functionalities.

## Introduction

Global climate change directly influences crop production systems for food (Wheeler and Von Braun, 2013). Biotic stress (pests and pathogens), abiotic stress (drought, heat waves, cold snaps, and flooding) and a combination of many of these factors occurring together (multifactorial stress combination) have devastating effects on crop growth and yield (Zandalinas et al., 2021). We need to develop healthy crop plants able to cope with environmental stress factors and to build sustainable agricultural systems to meet the food security needs in the increasing world population (Raza et al., 2025). Conventional crop breeding usually takes several years, whereas new speed-breeding methods would potentially achieve four to six generations per year in wheat, barley, chickpea and pea (Watson et al., 2018). A combination of speed breeding and nanoparticle-delivered CRISPR reagents has been proposed to achieve speedy crop enhancement within 1 year (Ahmar et al., 2021). However, whether CRISPR-edited crops are considered genetically modified (GM) or non-GM is still a conundrum and depends on regional regulation and public perception (Ahmad et al., 2023). Rather than growing climate-smart crops on the field, modern agriculture in most areas still heavily relies on the utilization of conventional agrochemicals to maintain crop health.

Emerging synthetic biology research has prompted the use of small technologies such as RNA interference (RNAi), short peptides and nanotechnology to generate novel agrochemicals for improving crop productivity and stress resilience (Rosa et al., 2022; Wang et al., 2022). RNAi is an effective gene silencing technique that can be applied in both temporal and permanent genetic modifications for disease control (Cai et al., 2018; Ahmad et al., 2025). Peptides released from pathogen effectors, host plants, and beneficial microbes can be recognized by specific plant receptors to trigger downstream signaling pathways involved in defense responses, growth and reproductive development and symbiosis (Stintzi and Schaller, 2022; Liu et al., 2024b; Song et al., 2025). Nanomaterials are useful to reduce inefficient overuse of pesticides (i.e., nanopesticides) and to release micronutrients or antibiotics in a controlled manner (i.e., nanofertilizers) for improving crop yield. With their small size, they can cross plant cuticle barriers, diffusing into the vasculature to deliver active ingredients efficiently and precisely (Kah et al., 2019; Lowry et al., 2019; Wang et al., 2022). Recent review articles describing current advances in the three small technologies used for sustainable agriculture are summarized in **Table 1**.

**Table 1:** List of the recent review articles regarding the current advances in three small technologies, nanotechnology, RNAs, and peptides.

Technology	Main focus and relevant examples in the review articles	Refs
Nanotechnology	A comprehensive analysis of the key properties of nanopesticides compared with their non-nanoscale analogues is provided. 1,163 nanopesticides are classified into 15 types. The anti-microbial mechanisms and modes of action in planta are revealed.	(Wang et al., 2022)
	Applications of nanopesticides and nanofertilizers for seed treatment, their associated challenges and risks assessment methodologies are discussed. 12 inorganic and organic nanotechnology-based agrochemicals used for seed priming, coating, and pelleting are compared.	(Shelar et al., 2023)
	A perspective regarding an eco-friendly and sustainable solution by using bionanofertilizers consist of nanomaterials and beneficial microorganisms for crop management and soil health is provided. The growth impacts and crop targets of 37 nanofertilizers and 17 bionanofertilizers are compared.	(Arora et al., 2024)
	The nano-enabled mechanisms of immunomodulation against plant pathogens are discussed. 26 cases of engineered nanomaterials used for enhancing disease resilience in agricultural crops are compared.	(Masood et al., 2024)
	The understanding of nanocarrier properties and their interactions with plant cells is highlighted for enhancing efficiency and efficacy of nano-based agrochemicals. Targeted delivery of nanoparticles into various plant subcellular compartments is summarized.	(Lowry et al., 2024)
RNAs	Nanotechnology-based delivery systems of genes and pesticides are discussed. 21 nanoparticle-mediated RNA pesticides for pest control and plant disease management are compared.	(Li et al., 2023)
	RNA-based active ingredients as innovative agrochemical are highlighted. Field-trail cases of RNA-based biocontrols against the Colorado potato beetle, corn rootworm, and soy stink bug are discussed.	(Bramlett et al., 2020)
Peptides	The use of RNAs and peptides as alternatives to current contentious fungicides is presented. 13 short peptides and 12 dsRNAs against relevant fungal and oomycete pathogens of crop are compared.	(Rosa et al., 2022)
	Web resources of natural agents with potential pest and pathogen control are surveyed. Databases and prediction servers of natural products and antimicrobial peptides are compared.	(Jin et al., 2021)
	The action mechanism of plant-derived antimicrobial peptides (AMPs) toward phytopathogens and human pathogens is discussed. 50 plant-derived AMPs with experimentally validated antimicrobial effects active against pathogens and abiotic stresses are compared.	(Ghanbarzadeh et al., 2024)

These articles provide a comprehensive overview and a balanced perspective when applying the three small technologies into sustainable agriculture.

Intrinsically disordered proteins/regions (IDPs/IDRs) may be the key molecules for developing innovative agrochemicals for sustainable agriculture. They are a group of proteins or amino acid regions without fixed 3D structures. Their structural flexibility enables them to quickly respond to environmental changes and cellular chemistry and interact with multiple binding partners to serve as regulatory hubs in various biological processes (Haynes et al., 2006; Uversky, 2009, 2019; Moses et al., 2023). IDPs/IDRs are key triggers of liquid–liquid phase separation (LLPS) to form biomolecular condensates (also known as membrane-less organelles/assemblies) in a spatiotemporal manner. This process is essential for regulating gene expression, the sequestration of specific factors in cellular programming, and the interconnection between diseases and immunity (Uversky, 2017; Boccaccio et al., 2023; Hirose et al., 2023). The diverse condensates formed via multivalent RNA–RNA, RNA–protein, and protein–protein interactions between ribonucleoproteins (RNPs) are called RNP granules. They have drawn significant attention because their dysfunction leads to cancers, neurodegenerative disorders and viral infections (An et al., 2021; Ripin and Parker, 2023).

Although understanding condensate formation provides new insights into human diseases and novel therapeutic opportunities (Mitrea et al., 2022), their physiological relevance should not be neglected (Guan et al., 2025). In plants, protein disorder has versatile roles in regulating hormone signaling, developmental processes and stress responses (Hsiao, 2022, 2024; Wu and Li, 2024). Plant IDPs/IDRs are involved in machineries that regulate transcriptional and post-transcriptional gene silencing via binding to RNA and serving as scaffolds for various regulatory condensates. These condensates are essential for controlling flowering time, a key agronomic trait affecting crop yield and quality (Liu et al., 2024a; Shang et al., 2024). They are also hijacked targets during virus infection (Li et al., 2021b; Liu et al., 2024a). Phytohormones such as salicylic acid, abscisic acid (ABA), and jasmonic acid control plant stress responses, whereas reactive oxygen species (ROS) are involved in systemic signaling (Myers et al., 2023; Das et al., 2025; Khan, 2025). Stress-responsive IDPs are often regulated by these phytohormones and also serve as stress sensors by forming condensates to fine-tune signaling or stress granules to balance the storage, translation, and degradation of RNA (Hsiao, 2022, 2024; Peng et al., 2025). A well-known group of IDPs is Late Embryogenesis Abundant (LEA) proteins, which are highly expressed in plant seeds before they enter the desiccation phase and confer multiple abiotic stress tolerance (Hernández-Sánchez et al., 2022; Hsiao, 2024). Recent examples are tomato bushy stunt virus hijacking the host sumoylation machinery to form condensate structures for viral replication (Lin and Nagy, 2025); rice IDR-mediated stress granule formation protecting mRNAs of *OsNCED4*, a key gene for ABA biosynthesis, from degradation under drought stress (Wang et al., 2024); and the pathogen-induced long noncoding RNA *ALEX1* regulating IDR-mediated transcription factor ARF3 condensation and complex assembly for modulating the jasmonic acid signaling pathway in rice bacterial blight resistance (Lei et al., 2025). These examples highlight the importance of plant protein disorder for switching on/off stress signaling. With knowledge of current advances in the three small technologies (RNAs, peptides and nanotechnology), this article aims to discuss how protein disorder can assist in agrochemical innovation to improve crop health.

## Small RNAs and short peptides lead to big impacts

A substitute for conventional pesticidal agrochemicals (insecticides, herbicides, and fungicides), which have unfavorable impacts on the agricultural ecosystem and human health, is the use of low-risk biomolecules based on small RNAs and short peptides (Rosa et al., 2022). RNAi is an evolutionarily conserved mechanism in eukaryotes silencing the expression of endogenous genes or genes of pathogens and pests at the (post-)transcriptional level (Kuo and Falk, 2020; Taning et al., 2020; Krzyszton et al., 2025). With its sequence-specific targeting, RNAi is unique in selectivity and efficiency as compared with other conventional agrochemicals and can be applied in transgenic or transient ways such as spray-induced gene silencing (SIGS) or root/seed soaking (Bramlett et al., 2020; Taning et al., 2020). Oomycete and fungus *Dicer-like 1* and *2* and cytoskeleton-associated genes such as *Dynactin* and *Suppressor of actin* represent promising pathogen targets via both transgenic approaches and SIGS, applied before pathogen infection in various fruit and vegetable crop plants (Wang et al., 2016b; Qiao et al., 2021). RNAi-based GM plants benefit from its systemic presence and season-long effect, which is unobtainable with external application so far. SmartStax PRO is the first corn trait to use RNAi to target the *Sucrose non-fermenting 7* gene in western corn rootworm for pest control (Head et al., 2017; Reinders et al., 2023). The major benefits of *ex planta* application include the quick response to changing pest pressure, the adaptation of formulation and application rates, and the use when a GM route is not feasible or acceptable (Bramlett et al., 2020). Although RNAi efficacy may be increased by chemically modified double-stranded RNA (dsRNA) for greater resistance to soil and insect saliva nucleases (Howard et al., 2022), a study shows that high pressure-sprayed naked dsRNA does not silence a reporter gene in transgenic *Nicotiana benthamiana* plants (Uslu et al., 2020). This finding raises concerns about the efficiency of RNAi-based agrochemical formulation and its delivery in host plants, which will be discussed in a later section.

Antimicrobial peptides (AMPs) are generally produced by all eukaryotic organisms with various anti-pathogen activity, immunomodulatory action, amino acid composition, and structural characteristics (Wang et al., 2016a; Kang et al., 2019; Zhang et al., 2021b). Plant-derived AMPs can be classified into defensins, nodule-specific cysteine-rich (NCR) peptides, lipid transfer proteins, α-hairpinins, knottin, thionin, cyclotide, etc., which are expressed under biotic and abiotic stress (Ghanbarzadeh et al., 2024). Recent examples show that the spray application of a *Medicago truncatula* defensin MtDef4 variant and a symbiotic peptide NCR044 can reduce gray mold disease symptoms caused by *Botrytis cinerea* in tomato and tobacco (Velivelli et al., 2020; Li et al., 2024), thus highlighting the use of plant-derived AMPs as potential biofungicides. Of note, knottin Pea Albumin 1, subunit b (PA1b) and its *Medicago* homologue AG41 show insecticidal activity against several pests such as aphids and weevils via binding to ATPase in the insect midgut for the formation of apoptosis bodies (Chouabe et al., 2011; Muench et al., 2014; Eyraud et al., 2017; Diya et al., 2023). Testing such insecticide activity on crop plants is needed. The ring structure of cyclotide and thionin endows them with high resistance to thermal and chemical denaturation as well as proteolytic degradation, for potentially durable biopesticides (Li et al., 2021a; Saberi Riseh et al., 2025). A recent study uses cellulose synthase 2, the *Plasmopara* oomycete cell wall biosynthesis enzyme, as a bait to screen a combinatorial 8-amino acid peptide library (Colombo et al., 2020). The authors identify a synthetic peptide aptamer able to prevent grapevine downy mildew and potato late blight diseases, which shares multiple AMP features (Colombo et al., 2020), suggesting its potential application in various crop diseases. Moreover, synthetic peptides may be used as crucial signaling molecules to attract beneficial microbes and deter pathogens to form ideal synthetic microbial communities (Yusuf et al., 2025a). Novel strategies are useful to rationally design and synthesize peptide-based agrochemicals. Such strategies include using amino acid residue substitution to enhance the antimicrobial efficacy of AMPs (Ting et al., 2020), screening combinatory libraries to select short peptides (Rosa et al., 2022), and identifying the associations between peptides and their corresponding targets of pathogens and plants via structural analysis and prediction tools (Gressel, 2022; Zhai et al., 2024; Adam et al., 2025; Jin et al., 2025).

## Nanotechnology improves agrochemicals

Nanotechnology is important for sustainable agriculture because the versatile physiochemical properties of nanomaterials can be engineered for different agrochemical purposes, as an intervention itself or a delivery platform for stable transformation or transient applications (Lowry et al., 2019, 2024; Wang et al., 2022). Nanoplatforms for the targeted delivery of RNAs and large CRISPR/Cas9 plasmids *in planta* are useful for altering genetic crops (Jo et al., 2020; Yan et al., 2022). Topical applications of layered double hydroxide clay nanosheets loaded with dsRNA prolong crop protection against viruses (Mitter et al., 2017), insects (Jain et al., 2022) and fungi (Niño-Sánchez et al., 2022). RNAs can fold into complex 3D architectures to achieve their versatile functions, which is inspiring the research of RNA-based nanoparticles for programmable immunomodulation in therapeutics (Chandler et al., 2021; Haseltine et al., 2025). The application of RNA-based nanoparticles to crop plants awaits invention. Nevertheless, nanofertilizers containing nutrient-rich elements have been used for seed priming and increasing crop productivity (Shelar et al., 2023). Manganese (Mn)-based nanomaterials with good ROS scavenging ability can be useful to improve cotton salt tolerance (Liu et al., 2023), which extends their application from pesticides to abiotic stress resilience of crops.

By manipulating the chemical composition, stimuli-responsive nanoparticles can respond to stimulus factors such as external pH, light, temperature, and pest enzymes to facilitate the targeted release of pesticides (Camara et al., 2019). pH-responsive nanopesticides can have higher release of pesticides in the alkaline environment of targeted insects but lower release in the acidic environments of beneficial insects such as honey bees and dew (Hou et al., 2023; Du et al., 2025). Although a controlled release of nanoparticles of carboxymethyl chitosan-modified carbon can enhance anti-UV properties for slow degradation and prolonged persistence (Song et al., 2019), UV and near-infrared light can also be stimuli to accelerate the release of nanopesticides (Liu et al., 2022; Zong et al., 2023). Besides responding to changing pH, dual- and triple-responsive nanopesticides have been designed to respond to temperature (Lin et al., 2024); insect enzymes such as laccase and esterase (Zhang et al., 2021a; Shan et al., 2023); and glutathione, a reducing agent widely present in insect cells (Zhao et al., 2022; Shan et al., 2023).

Engineering the multiple responsiveness of nanoparticles is useful to cope with the multifactorial stresses that crop plants are facing (Zandalinas et al., 2021). The translocation of nanoparticles from the cytosol into different subcellular compartments can be engineered by means of various transit peptides and surface moieties (Sperschneider et al., 2017; Lowry et al., 2024), which highlights the further improvement of spatial controlled release. A recent study shows that a novel cationic symmetrical peptide with anti-citrus pathogenic bacteria activity can self-assemble to form nanoparticles, which suggests that peptides can be potential constituents of nano-systems (Shuai et al., 2019). The highly tunable features of composition and flexible shape and surface chemistry of nanomaterials is similar to the rationale of IDPs/IDRs. Indeed, the disorder-to-order transitions of IDPs inspire the development of engineered biomimetic DNA machines by assembling disordered morphologies into ordered triangular architectures (Wang et al., 2025). Stress-sensing, self-assembly and molecular shielding features of protein disorder are expected to shape the fabrication of nanotechnology-based agrochemicals in terms of spatial-temporal controlled release and selective targeting to better tackle environmental stresses in a rapidly changing climate.

Green synthesis via microorganisms is a promising way to produce innovative agrochemicals (Arora et al., 2024). Recent studies in watermelon show that biogenic copper nanoparticles prevent bacterial fruit blotch and bio-functionalized manganese nanoparticles suppress Fusarium wilt; both metal-based nanoparticles are generated by the rhizosphere soil bacteria *Bacillus* (Noman et al., 2023b, 2023a). Hence, more beneficial microbes are expected to be used for the production of nanotechnology-based agrochemicals with potential scale-up via fermentation.

## Agrochemical innovation for/via dynamic disorder

Although current (nano)pesticides have a specific target such as an enzyme of pathogens, the case of using a sugar signaling molecule analog DMNB-T6P as a biostimulant to improve wheat yield highlights an idea of agrochemical innovation to target a whole pathway (Griffiths et al., 2025). Examples are master regulators in transcription/translation controlling events or tunable switches triggering phase separation and downstream signaling pathways, which are critical for crop productivity and stress responses or pathogen virulence. IDPs/IDRs and their corresponding condensates are proposed to be candidate targets of innovative agrochemicals. Several cases are shown in non-plant systems such as synthetic condensates created by RNP-IDP fusion enabling the modulation of cell function via translational enhancement in *E. coli* (Shapiro et al., 2025); designed binders targeting IDPs/IDRs to disrupt or inhibit the formation of stress granules or pathogenic amyloid structures in human HeLa cells (Liu et al., 2025); and a synthetic hairpin RNA incorporating a phage coat protein with IDRs to form programmable RNP granules in *E. coli* (Granik et al., 2022, 2025). In the following paragraphs, I present some thoughts on incorporating the three small technologies to target specific condensates in plant cells for desired agronomic traits such as yield and stress resistance.

Biomolecular condensates may be a prerequisite for anhydrobiosis, life without water (Elder et al., 2025), as suggested by the brine shrimp LEA6 protein undergoing LLPS to form a glassy state for desiccation tolerance (Belott et al., 2020). This trait is shared by rare organisms spanning every kingdom of life, including orthodox seeds in plants (Boothby and Pielak, 2017). Studies *in planta* show that Arabidopsis LEA6-2.1 is important in maintaining the glassy state and longevity of seeds for water deficit tolerance (Arroyo-Mosso et al., 2025). Also, disordered rice RePRP presented a novel root-to-seed transition mechanism for adaptation under water deficit (Hsiao et al., 2020; Hsiao, 2022), suggesting that the anhydrobiotic glassy state observed in orthodox seeds may be critical for desiccation tolerance. The IDR of the core protein kinase SnRK2 prompts condensate formation under severe hyperosmotic stress and initiates downstream ABA signaling by spatial segregation (Yuan and Zhao, 2025). Taken together, IDP/IDR-mediated biomolecular condensates are key regulators in plant drought responses. Therefore, agrochemical innovation can be proposed to engineer condensate formation and dynamic LLPS processes and trigger a temporary anhydrobiotic glassy state to help crops pass through dry periods via the small technologies RNAs, peptides and nanotechnology.

The race of revealing protein disorder between plant hosts and pathogens is continuing (Hsiao, 2024; Mughal and Caetano-Anollés, 2025). Current research into AMPs and nanopesticides focuses on pathogenic microbes and insect pests, but more *in planta* experiments on the field are necessary. In human disease, LLPS is a promising target for treating bacterial infection as well as therapeutic intervention in viral infection (Yusuf et al., 2025b). Although the idea of synthetic biomolecular condensates is recently tested *in planta* to limit the accumulation of symptoms of tobacco mosaic virus (Stanfield and May, 2025), more research is required to prove this concept. Understanding the molecular mechanism via biophysical characterizations of LLPS and IDP/IDR-mediated condensate formation and manipulating the physiochemical features of the small technologies RNAs, peptides and nanotechnology will help in engineering synthetic condensates as well as generating more precise and sophisticated agrochemicals.

Protein disorder is not promiscuous; the dynamic behaviors of IDPs/IDRs are governed by physicochemical properties of their amino acid sequences. Several studies have explored the sequence-encoded molecular grammar of IDPs/IDRs (Hoffmann et al., 2025; Vashishtha and Sabari, 2025). Rapid nanoscale IDP/IDR dynamics determines the mesoscale physical characteristics of condensates, such as their viscosity and molecular transport (Galvanetto et al., 2025). Understanding the principles of dynamic protein disorder and LLPS is necessary to precisely design agrochemicals to navigate pivotal physiological processes and stress signaling for improving crop health. The development of pharmaceuticals for neurodegenerative diseases has focused on inhibiting pathogenic condensate formation (Mitrea et al., 2022; Visser et al., 2024), such as small-molecule dissolution of stress granules (Uechi et al., 2025) and different nanoplatforms in preventing IDP aggregation (Lo et al., 2025). However, research into agrochemical innovation should proceed bidirectionally to both dissolve and promote condensate formation *in planta* and pathogens depending on various agronomic purposes. Protein disorder has been considered undruggable by traditional methods, but innovative approaches in therapeutical research strive to overcome the obstacles (Lazar et al., 2025; Sun et al., 2025). Such examples are adaptive peptides selectively antagonizing the pathogenic effects of IDPs without affecting their physiological functions (Fantini et al., 2025) and computational-designed binders targeting pathogenic IDRs with high binding affinity and experimental success (Wu et al., 2025). Hence, mechanistic investigation of multivalent interactions *in vivo* and physicochemical characterizations of protein disorder *in vitro* will facilitate future agrochemical innovation via efficient computational tools and template library screening.

## Intelligent agrochemicals for crop health in the future

The controversies and conundrum of GM and gene-editing crops are ongoing (Ahmad et al., 2023), and modified crops take time to be commercialized. Golden rice, enriching rice grains with beta-carotene to address the public health concern of vitamin A deficiency, was generated 20 years ago, but its commercialization is still a challenge (Ye et al., 2000). The closure of the leading Canadian biotech company Medicago Inc., producer of Covifenz (SARS-CoV-2 virus-like particle vaccine), is another example of commercialization difficulties (Benvenuto et al., 2023). Instead of using plants as a molecular farm producing pharmaceuticals (Zahmanova et al., 2023), information obtained from biomedical research that have been established and safely used in mammalian systems should be applied to improve agrochemical development. Genetic tools are important for understanding the mechanism of various crop plant physiology and stress responses, which offers a knowledge base for agrochemical invention. Besides striving to generate climate-smart crops that may face multiple challenges and restrictions, an alternative is to develop sustainable agrochemicals to quickly and temporarily shape crop physiology in response to rapidly evolved pathogens and drastic climate changes.

**Figure 1:**
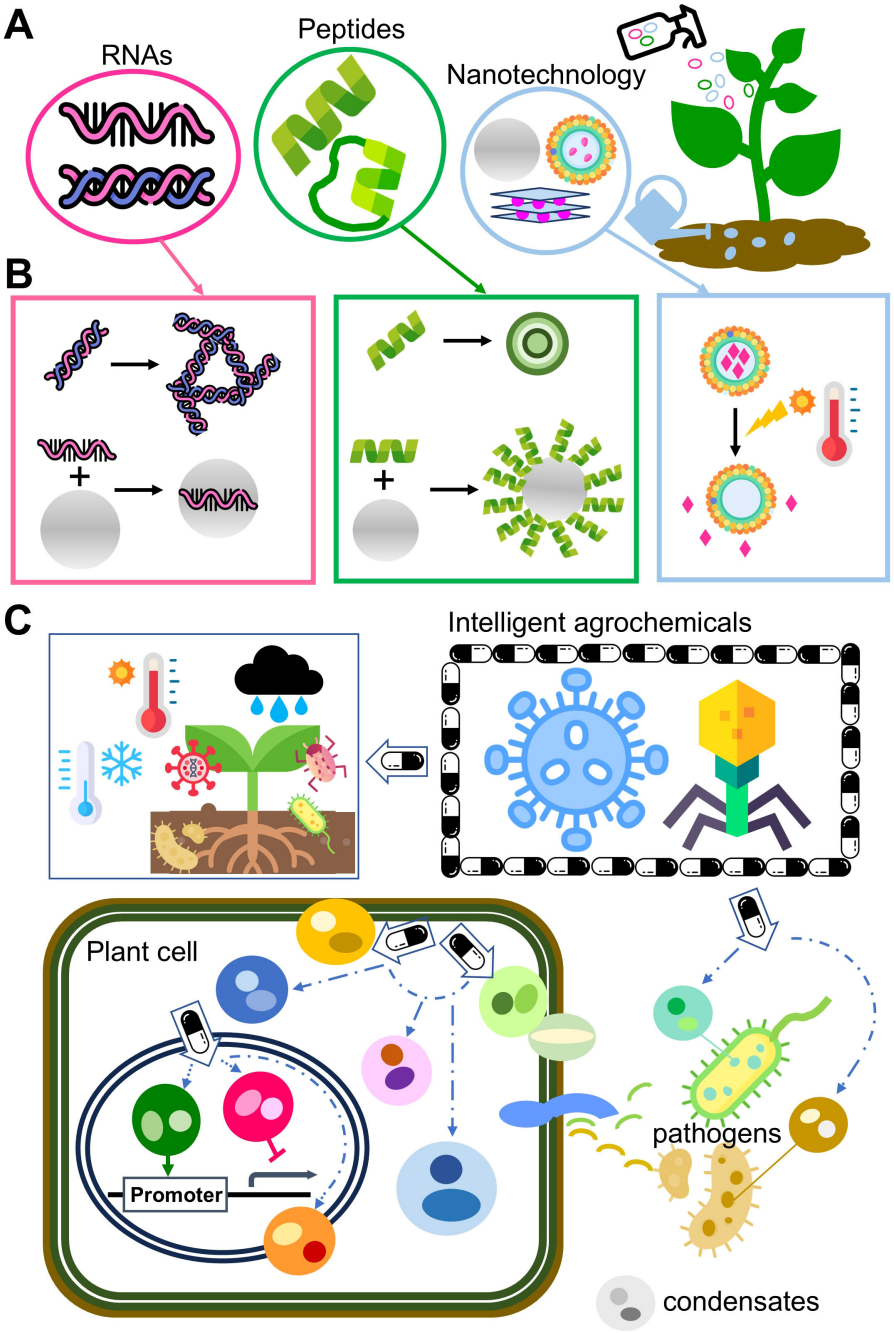
Agrochemical innovation for improving crop health. **(A)** The current advances in the three small technologies mentioned in this article, RNAs, peptides and nanotechnology, used as pesticides, fertilizers or genetic engineering aids. RNAs can be used for silencing key genes of pathogens, peptides can serve as toxins or signaling molecules to infer pathogens, and nanomaterials can be used directly as nanopesticides/nanofertilizers or serve as nanocarriers to encapsulate active ingredients. **(B)** Proposed engineering of the three small technologies for developing innovative agrochemicals with better efficiency and tailored functionalities. Both RNAs and peptides can fold into complex 3D architectures to form RNA/peptide-based nanoparticles. Alternatively, RNAs/peptides can be formulated with various nanomaterials to form a chimera agrochemical. Nanopesticides can be engineered with stress responsiveness in spatial-temporal controlled release. **(C)** Future intelligent agrochemical innovation with specificity and precision. A prototype of an intelligent agrochemical will be a chimera molecular machinery of the three small technologies or a pseudo virus-like particle such as a disarmed virus vaccine. By incorporating the three small technologies, a group of future intelligent agrochemicals will be able to target protein disorder and condensate formation to switch on/off signaling and programming for regulating the development, immunity and stress resistance in crop plants; inhibiting growth and virulence in pathogens; and mediating interactions between plants and microbes.

Inspired by the concepts of intelligent proteins (Tripathi et al., 2025) and microscopic engineering vehicles (Shangguan et al., 2024), several “intelligent agrochemical” strategies to improve crop health are proposed (**Figure 1**): 1) nanoplatforms with responsiveness to various stimuli (combinatory stress pressures) and able to target various cellular compartments with controlled release; 2) nanoparticles formulated with RNAs and/or peptides with versatile tailored functionalities; and 3) the incorporation of 1) and 2) to switch on/off signaling and cellular programing via targeting IDPs/IDRs, LLPS and condensate formation. Resembling drug discovery in biomedical research, prototypes of intelligent agrochemicals will be a chimera molecular machinery or pseudo virus-like particles such as disarmed virus vaccines for improving crop health. Protein disorder is double-edged sword, and modern pharmaceutical studies are changing this challenge into chance for novel drug design. Enlightened by this innovation, future agrochemical development should move forward via current small technologies and protein disorder.

## Data Availability

The original contributions presented in the study are included in the article/supplementary material. Further inquiries can be directed to the corresponding author.
